# Isophorone derivatives as a new structural motif of aggregation pheromones in Curculionidae

**DOI:** 10.1038/s41598-018-37156-8

**Published:** 2019-01-28

**Authors:** Diogo Montes Vidal, Marcos Antonio Barbosa Moreira, Miryan Denise Araujo Coracini, Paulo Henrique Gorgatti Zarbin

**Affiliations:** 1 Departamento de Química - Laboratório de Semioquímicos, Universidade Federal do Paraná, Curitiba, PR Brazil; 2 Departamento de Química, Universidade Federal de Minas Gerais, Belo Horizonte, MG Brazil; 3 Embrapa Tabuleiros Costeiros, Aracaju, SE Brazil; 4 Centro de Ciências Biológicas e da Saúde, Universidade Estadual do Oeste do Paraná, Cascavel, PR Brazil

## Abstract

The beetle *Homalinotus depressus* (Coleoptera: Curculionidae) is a major pest of coconuts in the Northern region of Brazil, for which environmentally friendly methods of control are desired. Behavioral responses of *H*. *depressus* to airborne volatile extracts from conspecifics suggested the presence of a male-produced aggregation pheromone. GC analyses of these extracts showed the presence of four male-specific compounds. Analytical data in combination with the synthesis of standards led to the identification of the male-released semiochemicals as epoxyisophorone (**1**), isophorone (**2**), homalinol (**3**), and 2-hydroxyisophorone (**4**), of which (**3**) was the major constituent. The configuration of homalinol was determined to be *cis* on the basis of retention times of synthetic *cis* and *trans* synthetic standards. Enantiomers of *cis*-homalinol were obtained in high enantiomeric excess by using biocatalysis. Their separation on a GC enantioselective column (β-Dex325®), allowed us to unambiguously determine that the absolute configuration of natural homalinol was (1*R*,2*R*,6*S*). Field bioassays demonstrated that both the synthetic major compound per se and mixtures of all four male-specific compounds were attractive to *H*. *depressus*.

## Introduction

Volatile organic compounds present in nature are produced and released by plants, animals, and microorganisms. Some of these compounds are produced as hormones, semiochemicals (including pheromones), as well as plant defense compounds. Other metabolites are merely biosynthetic byproducts^1^. Communication among insect involves various modalities^2^, including visual, vibrational, tactile, and chemical communication. Aggregation pheromones are sex specific pheromones which attracts both sexes of the species^3^. They have been identified in species from different insect Orders, *e*.*g*. Coleoptera, Diptera, Hemiptera, Dictyoptera, and Orthoptera^4–8^. Various insect species in the family Curculionidae (Coleoptera), are serious agriculture pests thus prompting a great deal of research^9–11^ aimed at finding new methods of control or attractants for trapping systems. Most of the aggregation pheromones identified in this group are from species in the genus *Rhynchophorus*^9^, *e*.*g*. rhynchophorol (6-methyl-2-(*E*)-hepten-4-ol) isolated from *R*. *palmarum*^12^. A complex aggregation pheromone system, (1*R*,2*S*)-grandisol and (1*R*,2*S*)-grandisyl acetate, was identified from the first species of *Homalinotus* genus studied, *Homalinotus validus*^13^. The chemical structures of the compounds described as aggregation pheromones of Curculionidae to date can be divided into two different classes. The first is related to mostly cyclic monoterpenoids, such as, grandisol, which was originally identified from *Anthonomus grandis*^14^. The second class is represented by short, and methyl branched alcohols, ketones, and esters, as for example 3-hydroxy-4-methyl-5-nonanone, identified as a component of *Metamasius hemipterus* pheromone^15^.

Coconut (*Cocos nucifera*, Arecaceae) is one of the main Brazilian fruit crops, with social and economic importance due to the diverse range of products obtained *e*.*g*. coconut water, coconut milk, wood, fibers, biofuel, animal food, and oils, among others derivatives useful to the agro-industry. Pest insects cause significant losses to worldwide coconut industry^16^. Pest outbreaks are favored by plant intrinsic factors, such as continuous production of leaves and inflorescences, or extrinsic factors, including temperature, humidity, poorly conducted cultural dealings, and excessive use of pesticides leading to reduced populations of natural enemies^16^.

*Homalinotus depressus* (Coleoptera: Curculionidae) occurs in the Antilles, Brazil, Colombia, French Guiana, and Suriname^17^. *H*. *depressus* larvae construct galleries inside coconut trees, floral peduncle and stipe, thus interrupting the sap flow and promoting failing of flowers and fruits. In 2008, *H*. *depressus* was reported for the first time as a coconut pest in the Brazilian northern region^18^. Management of *H*. *depressus* populations is largely based on applications of carbamates or organophosphates. Due to the lack of lures for trapping adult beetles, population densities are estimated by manual collections of beetles in the field. Therefore, semiochemicals, such as sex and/or aggregation pheromones, are highly desirable for trapping and possibly use in direct control strategies (*e*.*g*., mass trapping^19^, attraction-and-kill^20^, and mating disruption^21^. Here, we describe the isolation, identification, and field evaluation of an aggregation pheromone produced by *H*. *depressus*.

## Results and Discussion

### Initial GC analyses and olfactory bioassays

**Figure 1:**
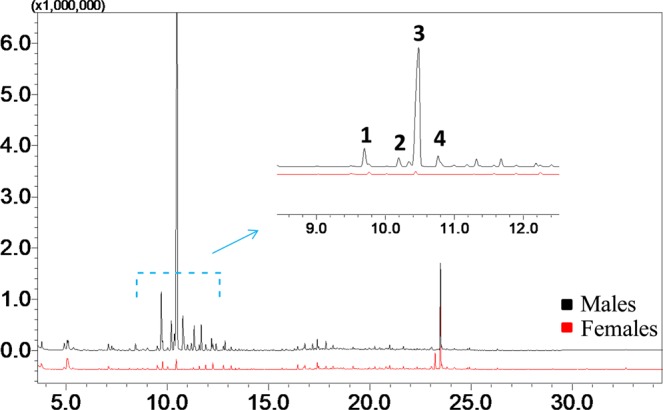
Total ion chromatograms of headspace extracts obtained from *Homalinotus depressus* males and females. **1**–**4**: male specific volatiles.

Volatile compounds emitted by *H*. *depressus* (kept under laboratory conditions) were collected by aeration and the GC comparison of aeration extracts from males to similar extracts from females showed the presence of four male-specific compounds (Fig. 1).

**Figure 2:**
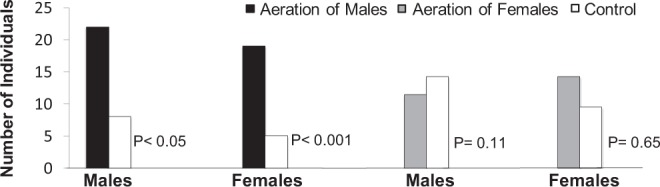
Behavioral responses of *Homalinotus depressus* to headspace extracts from both sexes. Statistical difference calculated according to binomial distribution test (p < 0.05, chi-square).

Y-tube olfactometer assays, performed with adults of both sexes, showed that volatile compounds from males were attractive to both sexes. By contrast, response of both males and females to airborne volatile collections from females did not differ significantly from the response to control (solvent only) (Fig. 2). We, therefore, surmised that the male-specific compounds are constituents of a sex aggregation pheromone and, consequently, pursued their identification.

Retention indexes^22^ (RIs) for the four natural products were calculated on RTX-5 (**1**–1096, **2**–1119, **3**–1127, **4**–1158) and EC-WAX (**1**–1540, **2**–1584, **3**–1789, **4**–1660) capillary columns.

### Structure assignment of compound 2

**Figure 3:**
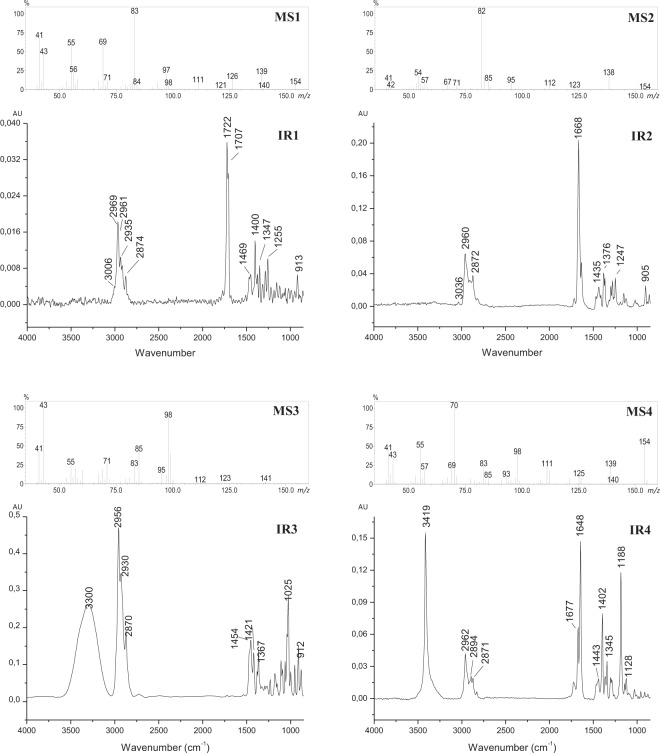
Mass and infrared spectra of the naturally occurring compounds **1**–**4** from *Homalinotus depressus* males extracts. Mass spectra: MS1) epoxyisophorone (**1**), MS2) isophorone (**2**), MS3) homalinol (**3**), MS4) 2-hydroxyisophorone (**4**). Infrared spectra: IR1) epoxyisophorone (**1**), IR1) isophorone (**2**), IR3) homalinol (**3**), IR4) 2-hydroxyisophorone (**4**).

The mass spectrum for compound **2** showed a base peak at *m*/*z* 82, and a possible M^+^ at *m*/*z* 138 (Fig. 3, MS2). Its infrared spectrum suggests a trisubstituted alkene due to the C=C stretching (1640 cm^−1^) associated with a band at 3036 cm^−1^ (C-H stretching on alkenes) (Fig. 3, IR2). Two relatively intense bands were observed at 2959 and 2941 cm^−1^, due to asymmetric and symmetric C-H stretching from CH_3_, thus indicating the occurrence of a branched CH_3_ moiety. An intense band was additionally observed at 1668 cm^−1^, suggesting the presence of an *α*,*β*-unsaturated ketone. Both IR^23^ and mass^24^ spectra were very similar to the spectra of 3,5,5-trimethylcyclohex-2-enone (isophorone). Another similarity between isophorone and compound **2** was the RI (1117 for isophorone on a DB-5 column^24^. Isophorone was first described in insects as a component of the defensive froth emitted by the grasshopper *Romalea micropterais*^25^, and more recently described as an allelochemical eliciting oviposition repellence on The Navel Orangeworm (*Ameylois transitella*)^26^. The structure of **2** was subsequently confirmed to be isophorone by comparing its retention time, and mass and IR spectra with those of an authentic standard.

### Structure assignment and synthesis of compound 1

The mass spectrum of compound **1** suggested a structure closely related to isophorone, with a base peak occurring at *m*/*z* 83 and a putative M^+^ ion at *m*/*z* 154, indicating a C_9_H_14_O_2_ molecular formula (Fig. 3, MS1). IR spectrum for this compound contained a C=O stretching band at 1722 cm^−1^ and three bands related to an epoxide: 3006 cm^−1^ (C-H stretching on epoxide carbon) and the pair of bands 1255 and 913 cm^−1^ (symmetric and asymmetric stretching of epoxide ring)^27^ (Fig. 3, IR1). Based on these data, compound **1** was proposed to be 4,4,6-trimethyl-7-oxabicyclo[4.1.0]heptan-2-one (epoxyisophorone) and then confirmed by synthesis, specifically, oxidation of isophorone with H_2_O_2_^28^ in 89% yield.

### Structure assignment and synthesis of compound 3

**Figure 4:**
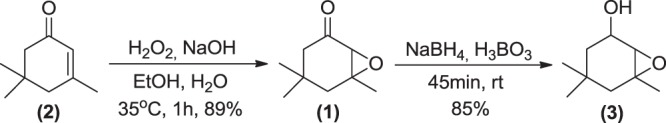
Synthesis of the racemate of epoxyisophorone (**1**) and a mixture of all the possible stereoisomers of homalinol (**3**), from isophorone (**2**).

The IR spectrum obtained from compound **3** showed O-H and a C-O stretching bands at 3300 and 1025 cm^−1^ respectively, suggesting the presence of a hydroxyl group, as well a band at 1025 cm^−1^ related to the axial deformation of C-O bond, indicating the presence of a hydroxyl in the structure (Fig. 3, IR3). The retention indexes (RI) calculated for this compound was 1127 on a RTX-5 column, and 1789 on an EC-WAX column. The difference between these two RIs is 656 units, while the difference expected for the presence of one hydroxyl group would be around 500^29^. These data suggest the contribution of an additional polar group in the structure. On the other hand, the mass spectrum for **3** showed a base peak at *m*/*z* 43, 141 as the highest *m*/*z* fragment, and 123 as a possible loss of water from 141 (Fig. 3, MS3). Additionally, the data suggested 141 as being related to the M^+^-15 and compound **3** as being a C_9_H_16_O_2_. Based on this dataset, the structure of **3** was proposed to be 4,4,6-trimethyl-7-oxa-bicyclo[4.1.0]heptan-2-ol. A mixture of all four possible stereoisomers in a ratio of 3:1 (*trans*:*cis*) was synthesized starting from **1**, by a solvent free reduction with NaBH_4_ in the presence of boric acid in 85% yield (Fig. 4)^30^.

Compound **3** is a novel natural product, being reported several times only as a synthetic derivative obtained during development of epoxidation or carbonyl reduction methodologies^31^. In this way, compound **3** was named “homalinol” derived from the *Homalinotus* genus.

### Relative and absolute configurations of homalinol (3)

**Figure 5:**
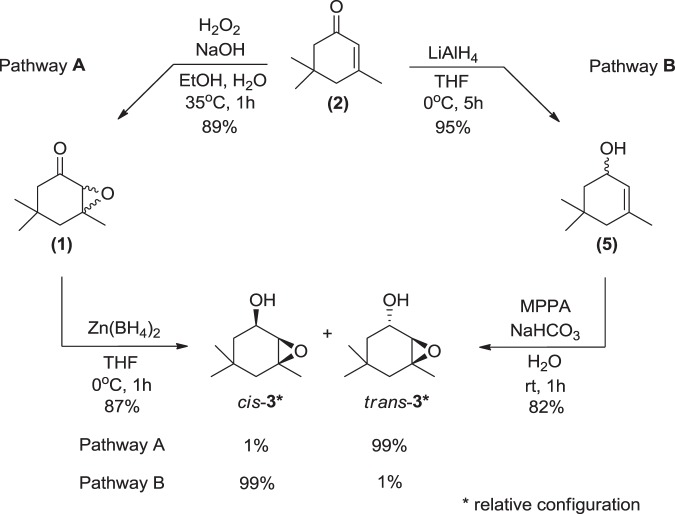
Synthesis of *cis*-**3** from pathway A: epoxidation of **2** followed by stereoselective reduction with zinc borohydride. Synthesis of *trans*-**3** from pathway B: reduction of the carbonyl of **2**, followed by stereoselective epoxidation of the double bond with MPPA.

The diversity of recognition forms of chirality by living organisms reveals the importance of the stereochemistry studies of semiochemicals, being a target of research on insect behavior over the past five decades^32^. The relative natural configuration of homalinol (**3**) was studied first by reducing epoxyisophorone (**1**) with freshly prepared zinc borohydride, producing *trans*-homalinol (*trans*-**3**) in 87% yield as a mixture of *trans*-**3**:*cis*-**3** (99:1) (Fig. 5, Pathway A). Zinc borohydride is widely used as a hydride source for reductions of *α* and *β*-hydroxyketones and *α*,*β*-epoxyketones due to the strong chelating effect of zinc^33^.

Racemate *cis*-**3** was obtained starting from the reduction of **2** to isophorol (**5**) with LiAlH_4_ in 95% yield followed by epoxidation with freshly prepared monoperphtalic acid (MPPA), resulting on a mixture of *cis*-**3**:*trans*-**3** (99:1) (Fig. 5, Pathway B)^34,35^. The stereoselectivity of this reaction can be rationalized as a mechanism correlated to that proposed for the Prilezhaev reaction, where the epoxidation occurs in a concerted manner, with interaction between the hydroxyl hydrogen from **5** and the carboxylic oxygen of the peroxyacid from MPPA^36^. The relative configuration of *trans*-**3** and *cis*-**3**, obtained stereoselectively as described above, was determined based on their respective NMR spectra and previously reported data^37,38^.

**Figure 6:**
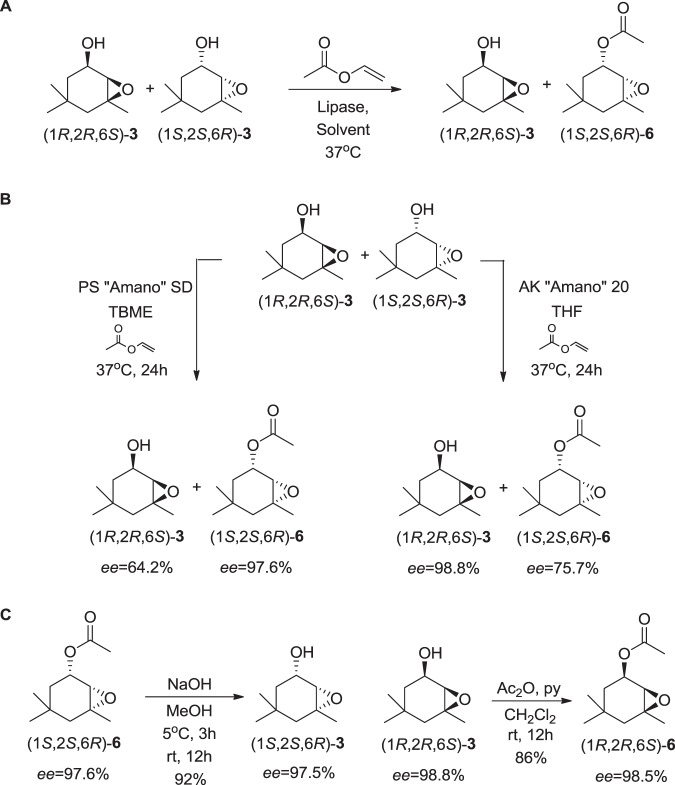
Enantioselective synthesis of *cis*-**3** and *cis*-**6** by biocatalytic approach. (**A**) Optimization of best conditions for kinetic resolution of *cis*-**3** were performed with five different lipases (CAL-B, PS “Amano” SD, AK “Amano” 20, AY “Amano” 30, and PPL) and three organic solvents (TBME, THF, and hexane). (**B**) The best conditions found in A were employed to synthesize (1*R*,2*R*,6*S*)-**3**, and (1*S*,2*S*,6*R*)-**6** with good enantiomeric excesses. (**C**) Synthesis of (1*S*,2*S*,6*R*)-**3** and (1*R*,2*R*,6*S*)-**6** by basic hydrolysis and acetylation, respectively.

Enantioselective GC analysis resulted in the resolution of the four stereoisomers of **3**, allowing us to determine that the natural compound from *H*. *depressus* is a single enantiomer of the *cis*-**3** mixture, (1*R*,2*R*,6*S*)-**3** or (1*S*,2*S*,6*R*)-**3** (Figure S1). Assays aiming the enantioselective synthesis of (1*R*,2*R*,6*S*)-**3** and (1*S*,2*S*,6*R*)-**3** were performed by kinetic resolution employing the lipases CAL-B (from *Candida antarctica*), AY “Amano” 30 (from *C*. *rugosa*), AK “Amano” 20 (from *Pseudomonas fluorescens*), PS “Amano” SD (from *Burkholderia cepacia*), and PPL (porcine pancreatic lipase) and four different organic media: TBME, hexane, vinyl acetate and THF. Vinyl acetate was used as acetate source in all tested conditions (Fig. 6A).

Results obtained by CAL-B catalysis were not satisfactory, given that good conversion rates but with low enantioselectivity were obtained. The best result using CAL-B was obtained with 24 hours of reaction with THF as solvent, with *ee*’s of 43.6% for the remaining alcohol and 60.8% for the acetate formed. Reactions employing AY “Amano” 30 and PPL were not satisfactory either, due to conversion rates (c) lower that 5% even after nine days of reaction. On the other hand, better results were found in trials employing PS “Amano” SD and AK “Amano” 20, as shown on Table S1. PS “Amano” SD assays resulted in excellent enantiomeric excesses for the acetate formed and consequently satisfactory enantiomeric ratios (*E*). It must be highlighted that the reaction employing PS “Amano”SD and TBME in 24 hours resulted in the acetylated product with *ee* 98,6% (c = 39,9%, *E* > 200). Reactions using AK “Amano” 20 resulted in excellent *ee* for the remaining alcohol with the best result found using THF in 24 hours, resulting in a mixture containing the alcohol with *ee* 99,9% (c = 55,5%, *E* = 65). PS “Amano” SD and AK “Amano” 20 were employed on preparative scale in order to obtain higher amounts of the acetylated product and the remaining alcohol respectively, as shown on Fig. 6B.

The only stereoisomer of **3** with absolute stereochemistry previously studied was (1*S*,2*S*,6*R*)-**3**, which was assigned by NMR of the Mosher’s ester derivative, where the specific rotation found was [α]_D_^24^ = −18.8 (c = 0.86, CHCl_3_, *ee* = 38%)^31^. The alcohol and acetate from each kinetic resolution were purified by flash chromatography in order to obtain samples of enantiomerically enriched compounds **3** and **6**. The specific rotation of [α]_D_^24^ = + 35.0 (c = 0.86, CHCl_3_, *ee* = 98.8%) was found for the remaining alcohol, confirming its absolute configuration as (1*R*,2*R*,6*S*). The alcohol (1*S*,2*S*,6*R*)-**3** was synthesized from the basic hydrolysis of (1*S*,2*S*,6*R*)-**6** in 92% yield (Fig. 6C), showing an [α]_D_^24^ = −34.5 (c = 0.86, CHCl_3_, *ee* = 97.5%).

This is the first case of description of compounds (1*S*,2*S*,6*R*)-**6** and (1*R*,2*R*,6*S*)-**6** as enantiomerically enriched compounds. The acetate (1*S*,2*S*,6*R*)-**6** showed an [α]_D_^24^ = −70.2 (c = 0.86, CHCl_3_, *ee* = 97.6%) after isolation from kinetic resolution. The (1*R*,2*R*,6*S*)-**6** enantiomer was obtained from (1*R*,2*R*,6*S*)-**3**, by treatment with acetic anhydride and pyridine in dichloromethane in 86% yield (Fig. 6C), showing an [α]_D_^24^ = +74.9 (c = 0.86, CHCl_3_, *ee* = 98.5%).

**Figure 7:**
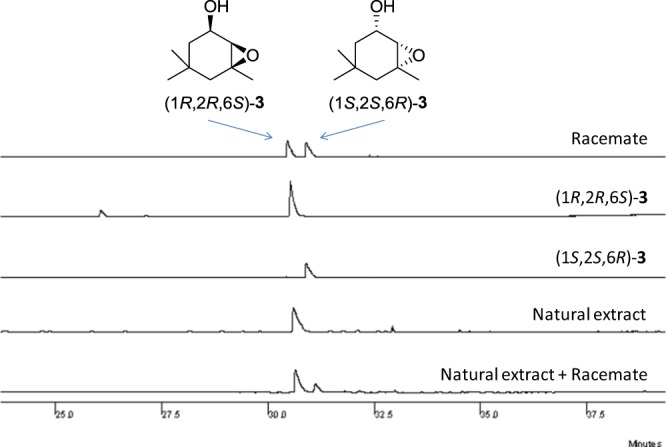
Comparison between the GC profiles of the racemate *cis*-**3**, (1*S*,2*S*,6*R*)-**3**, (1*R*,2*R*,6*S*)-**3**, the natural extract, and the coinjection of the racemate and the natural extract on a β-DEX 325® capillary column.

Figure 7 illustrates chromatograms obtained by GC employing a β-Dex 325® capillary column. Traces for the resolution of the racemate of *cis*-**3**, as well as the elution of enantiomers (1*R*,2*R*,6*S*)-**3** and (1*S*,2*S*,6*R*)-**3** are displayed. The analysis of the natural compound clearly demonstrated that it occurs as a single enantiomer which eluted with the same retention time of (1*R*,2*R*,6*S*)-**3**. Co-injection of (1*R*,2*R*,6*S*)-**3** with the natural extract confirmed that indeed (1*R*,2*R*,6*S*) is the absolute configuration of this natural pheromone.

### Absolute configuration of epoxyisophorone (1)

The determination of the absolute configuration of epoxyisophorone (**1**) released by *H*. *depressus* began with the oxidation of alcohols (1*S*,2*S*,6*R*)-**3** and (1*R*,2*R*,6*S*)-**3** using PCC, resulting in (1*R*,6*R*)-**1** (84% yield) and (1*S*,6*S*)-**1** (82% yield) respectively. The synthetic racemate **1**, the enantiomerically enriched (1*R*,6*R*)-**1** and (1*S*,6*S*)-**1**, and the natural compound **1** were analyzed on the β-Dex 325® capillary column, showing that the natural compound **1** is 1*S*,6*S*, as expected due to the orientation of the epoxide group in the major component (**3**) (Figure S2).

### Structure assignment of compound 4

**Figure 8:**
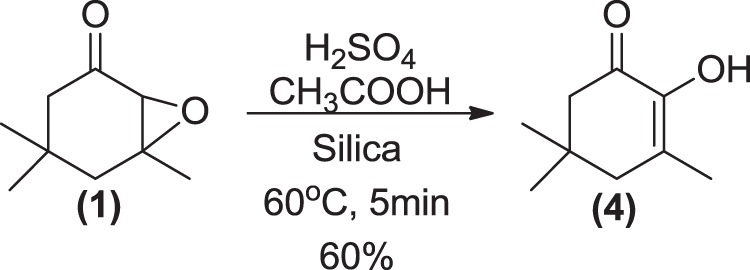
Synthesis of 2-hydroxyisophorone (**4**).

The IR spectrum of natural compound **4** (Fig. 3, IR4) showed an intense band at 3414 cm^−1^, characteristic of a hydroxyl group with a intramolecular hydrogen bond. A C=C stretching band was also observed at 1674 cm^−1^. On the other hand, no bands from hydrogen attached to *sp*^2^ carbons were observed, suggesting the presence of a tetrasubstituted double bond. Mass spectra obtained for this compound showed similarities with the spectra obtained for compounds **1**–**3**, a molecular ion peak at *m*/*z* 154 and the base peak at *m*/*z* 70 (Fig. 3, MS4). These spectral data were closely related to 2-hydroxy-3,5,5-trimethylcyclohex-2-enone (2-hydroxyisophorone), which was synthesized from (**1**), in 60% yield under solvent free conditions (Fig. 8). The synthetic standard coeluted with the natural compound, with identical mass and IR spectra, confirming the structure of the natural compound as the diosphenol 2-hydroxyisophorone (**4**). Compound **4** has been described previously as component of saffron (*Crocus sativus*)^39^ and different types of honey^40–42^. However, this is the first case of occurrence in animals.

### Laboratory and field bioassays using synthetic blends

Y-tube olfactometer bioassays performed with male and female *H*. *depressus* showed attractiveness of *cis*-**3** to both sexes, while *trans*-**3** and a mixture of *cis*-**3** and *trans*-**3** (1:1 w/w) significantly attracted only males when compared to hexane controls (Table S2).

**Figure 9:**
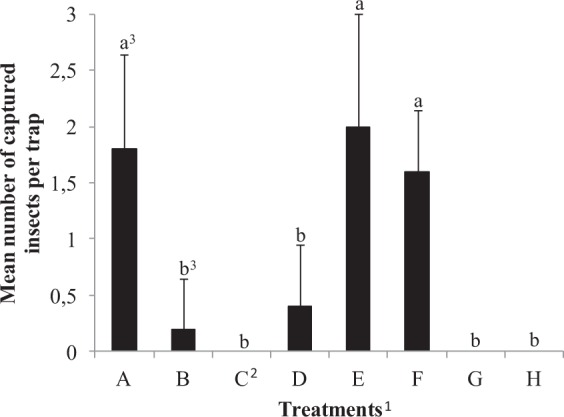
Mean numbers of insects captured in a field experiment testing different treatments, during a period of six days. Treatments: **A**, *cis*-**3**; **B**, *trans*-**3**; **C**, control; **D**, *cis*-**3** + *trans*-**3**; **E**, (*R*,*R*,*S*)-**3**; **F**, **1** + **2** + *cis*-**3** + **4**; **G**, **1**; **H**, **2**. ^1^A 2 × 5 cm piece of sugarcane was placed inside each trap^2^. The treatment labeled as control used an empty plastic bag as lure^3^. Different letters indicate statistical differences by ANOVA followed by Tukey test (p < 0.01, except for **A**–**D** and **B**–**F** where p < 0.05).

Figure 9 shows the field assay results, with the mean number of insects caught in each treatment. Traps containing only isophorone (**2**), epoxyisophorone (**1**), or hexane (control) did not catch any insects, while traps containing rubber septa with *trans*-**3**, *cis*-**3** + *trans*-**3** (1:1 w/w), or *cis*-**3** captured insects, but the results are not statistically different from treatments with no captures. Traps baited with *cis*-**3**, (1*R*,2*R*,6*S*)-**3**, or the complete mixture (*cis*-**3** + **1** + **2** + **4**) were significantly more attractive to insects than the other treatments but did not differ between themselves. These results showed that both, the racemate of the major component (*cis*-**3**), the pure naturally produced enantiomer ((1*R*,2*R*,6*S*)-**3**), and the mixture containing the four male-specific compounds were active under field conditions and may be used as lures for practical applications.

Although the field experiment showed that the male-specific compounds are bioactive on attractiveness of conspecifics, additional field experiments are needed, to optimize operational parameters such as dose/release rate, blend ratios, and trap type.

In summary, this study resulted in the identification, synthesis and field evaluation of four male specific compounds released by *H*. *depressus* and used as an aggregation pheromone. These compounds were identified as (1*S*,6*S*)-epoxyisophorone ((1*S*,6*S*)-**1**), isophorone (**2**), (1*R*,2*R*,6*S*)-homalinol ((1*R*,2*R*,6*S*)-**3**), and 2-hydroxy-3,5,5-trimethylcyclohex-2-en-1-one (**4**). These compounds represent a new class of structures for Curculionidae aggregation pheromones.

## Materials and Methods

### Insects

*Homalinotus depressus* pupal chambers and adults were collected in a commercial coconut farm (Socôco Farm – Mojú/PA, Brazil, −1.91, −48.76). Collected insects were sent to Laboratory of Semiochemicals at UFPR, Curitiba/PR-Brazil. Adults were sexed and maintained under laboratory conditions (28 °C, 85% RH, 12 h Light: 12 h Dark) and fed on sugarcane which was replaced every 72 hours.

### Volatile collection

Volatiles were collected by aeration method^43^. Five insects of each sex were conditioned separately inside of cylindrical chambers (30 cm (length) × 12 cm (ID)) containing a 10 × 2 × 2 cm piece of sugarcane. Charcoal filtered and humidified air flow (1 L.min^−1^) was applied inside the chambers in the upward direction. The released volatiles were absorbed by HayeSep D® filters (polydivinylbenzene, 795 m^2^.g^−1^, 80–100 mesh). Filters were extracted with hexane (200 µL) every 24 hours.

### Y-tube behavioral bioassays

The olfactometer consisted of a Y-shaped glass tube (4 × 40 cm) with two 20 cm arms. The odor sources, which consisted of pieces of filter paper (2 × 2 cm) impregnated with the synthetic compounds, natural extracts or hexane (control), were placed at the ends of the arms. An insect was introduced into the base of the olfactometer and the behavior of the insect was observed over 10 min. An insect walking against the air flow toward the odor source for more than 5 cm in any of the arms and remaining in the arm for more than 2 min was considered a response. An insect remaining in the main tube was considered to be no response. Each insect was tested only once, representing one replicate experiment. The odor source was replaced after every test. The experiments were performed between the 4^th^ and 6^th^ hours of the scotophase, the time of day of highest pheromone release. Insects that did not choose any of the arms were excluded from statistical analysis^44,45^. Data were analyzed using a chi-square test processed on BioEstat software (version 5.0).

Initially, four behavioral response experiments were performed; the responses of males to odors from males, males to odors from females, females to odors from males and females to odors from females. Each replicate was performed using 40 μL of extract of interest.

Bioassays conducted to observe the biological activity of the stereomers of the synthetic major male-specific compound were performed using 20 μL (50 ng.μL^−1^) of each standard.

### Gas chromatography coupled to mass spectometry (GC-MS)

GC-MS analysis were performed on a Shimadzu QP2010 Plus spectrometer, operating on electron impact mode (70 eV), coupled to a Shimadzu GC-2010. Samples were eluted on a RTX-5 (30 m × 0.25 mm × 0.25 μm; Restek Chromatography Products, USA), at 1 mL.min^−1^ (He). The initial column temperature was 50 °C, maintained during 1 minute and increased to 270 °C at a 7 °C per minute rate remaining at this temperature for 5 minutes.

### Gas chromatography coupled to infrared spectroscopy (GC-FTIR)

GC-FTIR analyses were performed on a Shimadzu GC-2010 coupled to a DiscovIR-GC (Spectra Analysis) detection system. The GC oven temperature was maintained at 100 °C during 1 minute, increased to 270 °C at 10 °C.min^−1^, and kept at this temperature for additional 10 minutes. Samples were eluted on a RTX-5 (30 m × 0.25 mm × 0.25 μm, Restek Chromatography Products, USA) at a 1 mL.min^−1^.

### Determination of retention indexes

Target analytes were coinjected with an *n*-alkanes series. The GC temperature started at 50 °C, and increased to 230 °C at a 3 °C/minute rate. Analyses were conducted using two different stationary phases, RTX-5 (30 m × 0.25 mm × 0.25 μm, Restek Chromatography Products, USA) and EC-WAX (30 m × 0.25 mm × 0.25 μm, GRACE, USA). Retention indexes were calculated based on van den Dool & Kratz method^22^.

### Enantioselective Gas Chromatography

GC analyses on enantioselective stationary phase were performed on a Varian 3800 gas chromatograph, equipped with a β-Dex 325 capillary column (25% 2,3-di-O-methyl-6-O-TBDMS-β-cyclodextrin, 30 m × 0.25 mm × 0.25 µm, Supelco, USA) and a FID detector system. GC oven initial temperature was 50 °C, maintained during one minute. After this time, the temperature was increased to 130 °C (3 °C.min^−1^), and later to 230 °C (7 °C.min^−1^). The temperature was maintained at 230 °C during 5 additional minutes.

### 4,4,6-Trimethyl-7-oxabicyclo[4.1.0]heptan-2-one (1)

A solution containing isophorone (**2**, 144.7 mmol, 20 g) in ethanol (250 mL), was added at 25 ^o^C to an aqueous solution of NaOH (620 mg in 20.5 mL of H_2_O). H_2_O_2_ (20.5 µL, 35%) was added to this solution during 20 minutes. The temperature was maintained between 30–35 °C during 1 hour. The resulting mixture was cooled to 25 °C, diluted with 170 mL of water, and the product extracted with CH_2_Cl_2_ (3 × 150 mL). The organic phase was washed with water, dried by Na_2_SO_4_, and the solvent evaporated by vacuum. The crude product was purified by flash chromatography (hexane/ethyl acetate: 9/1), resulting on isophorone oxide (**1**) in 89% yield (128.8 mmol, 19.85 g)^28^. ^1^*H NMR* (*200* *MHz*, *CDCl*_3_) *δ*: 0.82 (s, 3H), 0.96 (s, 3H), 1.34 (s, 3H), 1.56–1.66 (m, 1H), 1.67–1.76 (m, 1H), 1.93–2.06 (m, 1H), 2.44–2.59 (m, 1H), 2.96 (s, 1H). ^*13*^*C NMR* (*50* *MHz*, *CDCl*_3_) *δ*: 24.0, 27.8, 30.8, 36.2, 42.7, 47.9, 61.3, 64.3, 208.1. *MS* (*EI*, *70* *eV*)*: m/z* (*%*)*:* 154 (M^+^, 8), 139 (25), 126 (16), 111 (10), 98 (6), 97 (23), 84 (6), 83 (100), 82 (8), 81 (6), 79 (6), 70 (5), 69 (55), 67 (8), 58 (12), 57 (6), 56 (18), 55 (44), 53 (7), 43 (29), 42 (7), 41 (41). *IR* (*ZnSe*, *ν*_*max*_)*:* 913, 1255, 1347, 1400, 1469, 1707, 1722, 2874, 2961, 2969, 3006 cm^−1^.

### 4,4,6-Trimethyl-7-oxabicyclo[4.1.0]heptan-2-ol (3)

A mixture containing isophorone oxide (**1**) (100 mg, 0.65 mmol), NaBH_4_ (9 mg, 0.225 mmol), and boric acid (55 mg, 0.225 mmol) was macerated in a mortar with a pestle during 45 minutes. The reaction was quenched by adding a saturated solution of NaHCO_3_, followed by filtration. The resulting solution was extracted with CH_2_Cl_2_ (3 × 15 mL), and the solvent evaporated by vacuum. The crude product was purified by flash chromatography (hexane/ethyl acetate: 2/1), resulting on pure **3**, in 85% yield^30^. For NMR and MS data, see *trans*-**3** and *cis*-**3**.

### Zinc borohydride

To a magnetic stirred and dried 250 mL reaction flask under Ar atmosphere were added freshly melted^46^ ZnCl_2_ (4.180 g, 29.0 mmol) and NaBH_4_, (2.540 g, 67.8 mmol). THF (58 mL) was then added and the mixture was stirred under r.t. during 72 hours. The supernadant was employed on the reduction reactions, with an estimated hydride concentration of 4.4 mol.L^−1^ ^33^.

### trans-4,4,6-Trimethyl-7-oxabicyclo[4.1.0]heptan-2-ol (trans-3)

A solution containing **1** (5.00 g, 32.4 mmol) in THF (200 mL) was prepared and cooled to 0 °C. Freshly prepared Zn(BH_4_)_2_ solution (22.5 mL) was added and the resulting mixture stirred during 45 minutes at 0 °C. After this period, distilled water (100 mL) was added and the solution stirred for additional 30 minutes. The organic phase was isolated, dried with Na_2_SO_4_, and filtered. The solvent was removed by reduced pressure and the crude product purified by flash chromatography (hexane/ethyl acetate: 2:1), yielding *trans*-**3** in 85% yield (4.29 g, 27.5 mmol)^47^. ^*1*^*H NMR* (*400* *MHz*, *CDCl*_3_) *δ*: 0.91 (s, 3 H), 0.97 (s, 3 H), 1.17 (dd, *J* = 13.4 Hz, *J* = 7.4 Hz, 1H), 1.34 (s, 3H), 1.52 (d, *J* = 15.0 Hz, 1H), 1.64 (dd, *J* = 13.4 Hz, *J* = 5.8 Hz, 1H), 1.71 (d, *J* = 15.0 Hz, 1H), 2.33 (s, 1H), 2.96 (s, 1H), 4.15 (dd, *J* = 7.4 Hz, *J* = 5.8 Hz, 1H). ^13^*C NMR* (*100* *MHz*, *CDCl*_3_) *δ*: 24.1, 28.5, 28.8, 31.6, 42.4, 42.5, 59.5, 63.0, 65.8. *MS* (*EI*, *70* *eV*)*: m/z* (*%*)*:* 141 (M^+^-15, 4), 123 (7), 112 (7), 107 (7), 100 (8), 99 (41), 98 (92), 97 (21), 95 (27), 85 (65), 83 (36), 81 (12), 79 (12), 72 (10), 71 (32), 69 (30), 67 (21), 60 (18), 57 (24), 56 (12), 55 (33), 53 (11), 44 (4), 43 (100), 42 (6), 41 (45).

### 3,5,5-Trimethylcyclohex-2-enol (5) (Isophorol)

A suspension of LiAlH_4_ (15.4 mg, 400 mmol) in anhydrous THF (600 mL) was prepared and maintained at 0 °C, under argon atmosphere and magnetic stirring. Isophorone (**2**) (20 g, 142 mmol) was slowly added and the resulting mixture was gradually warmed to r.t. and stirred during 5 hours. After this period, the reaction was quenched by the slow addition of cold water (200 mL), followed by aqueous NaOH (15%, 100 mL), and by a second portion of cold water (200 mL). The resulting suspension was filtered under vacuum and the phases separated. The organic phase was dried over anhydrous Na_2_SO_4_ and concentrated under vacuum. The crude product was purified by flash chromatography (hexane/ethyl acetate: 8/2), yielding isophorol (**5**) (18.39 g, 131.4 mmol, 92%)^30,48^. ^*1*^*H NMR* (*200* *MHz*, *CDCl*_3_) *δ*: 0.88 (s, 3 H), 0.99 (s, 3 H), 1.16–1.29 (m, 1 H), 1.54–1.92 (m, 6 H), 4.14–4.30 (m, 1 H), 5.38–5.46 (m, 1 H). ^*13*^*C NMR* (*50* *MHz*, *CDCl*_3_) *δ*: 23.5, 26.2, 31.0, 31.2, 44.1, 45.2, 66.7, 123.7, 135.8. *MS* (*EI*, 70 *eV*)*: m/z* (*%*)*:* 140 (M^+^, 14), 126 (9), 125 (100), 122 (10), 107 (42), 105 (8), 93 (6), 91 (23), 84 (50), 83 (33), 82 (11), 81 (7), 79 (17), 77 (9), 69 (50), 67 (7), 65 (6), 57 (5), 56 (13), 55 (27), 53 (7), 43 (22), 41 (20).

### Monoperphtalic acid (MPPA)

Hydrogen peroxide (18.6 mL, 30%) was added at −5 °C under continuous stirring, to 68 mL of an aqueous solution of Na_2_CO_3_ (16.74 g, 134 mmol). The temperature was maintained between 0 and −5 °C, during the slow addition of phtalic anhydride (20 g, 134 mmol). After the addition of phtalic anhydride, the mixture was transferred to a separatory funnel containing ethyl ether (90 mL) and carefully acidified by the addition of an aqueous H_2_SO_4_ solution (8 mL of H_2_SO_4_ in 40 mL of H_2_O). The reaction was extracted with ethyl ether (3 × 30 mL), and the combined organic phase washed with ammonium sulfate (2 × 20 mL, 40%). The organic phase was dried with Na_2_SO_4_ during 12 hours, at −18 °C. The peracid concentration was determined by adding an aqueous solution of KI (30 mL, 20%) to the MPPA solution (2 mL). The released iodine was titrated after 10 minutes with a Na_2_S_2_O_3_ (0.1 mol.L^−1^)^34^.

### cis-4,4,6-Trimethyl-7-oxa-bicyclo[4.1.0]heptan-2-ol (cis-3)

Isophorol (**5**) (10 g, 71 mmol), was added to a mixture containing MPPA solution (153 mL, 85 mmol) and NaHCO_3_ aqueous solution (200 mL, 0.5 mol.L^−1^). The resulting solution was stirred at r.t. during 1 hour, followed by addition of brine (100 mL) and extraction with CH_2_Cl_2_ (4 × 100 mL). The combined organic phase was washed with brine, dried with anhydrous Na_2_SO_4_, and the solvent removed by vacuum. The crude product was purified by flash chromatography (hexane/ethyl acetate: 2/1), yielding *cis*-**3** in 84% yield (9.30 g, 59.64 mmol)^35^. ^*1*^*H NMR* (3*00* *MHz*, *CDCl*_*3*_) *δ*: 0.86 (s, 3H), 0.89 (s, 3H), 1.10–1.27 (m, 1H), 1.35 (s, 3H), 1.36–1.70 (m, 3H), 2.43 (s, 1H), 3.12–3.20 (m, 1H), 4.07 (ddd, *J* = 10.9 Hz, *J* = 6.1 Hz, *J* = 2.2 Hz, 1H). ^*13*^*C NMR* (*75* *MHz*, *CDCl*_3_) *δ*: 24.8, 26.5, 31.2, 31.3, 40.1, 42.3, 61.1, 62.2, 66.6. *MS* (*EI*, *70* *eV*)*: m/z* (*%*): 141 (M^+^-15, 4), 123 (5), 112 (4), 107 (5), 100 (7), 99 (40), 98 (88), 97 (13), 95 (19), 85 (50), 83 (25), 81 (9), 79 (9), 72 (8), 71 (30), 69 (22), 67 (16), 60 (16), 57 (21), 56 (9), 55 (25), 53 (8), 44 (6), 43 (100), 42 (6), 41 (42). *IR* (*ZnSe*, *ν*_*max*_): 912, 1025, 1367, 1421, 1454, 2870, 2930, 2956, 3300 cm^−1^.

### Kinetic enzymatic resolution of cis-3 – analytical scale

The enzymatic resolutions in analytical scale were performed employing six different lipases: CAL-B: Lipase B *Cândida Antarctica* (Novozymes, USA), AY Amano 30: Lipase *Cândida rugosa* (Amano, Japan), AK Amano 20: Lipase *Pseudomonas fluorescens* (Amano, Japan), PS Amano SD: Lipase *Burkholderia cepacia* (Amano, Japan), and porcine pancreas lípase (PPL) (Sigma-Aldrich, USA).

The apropriate organic solvent (1 mL of hexane, THF or TBME), the alcohol *cis*-**3** (30 µL, 0.17 mmol), vinyl acetate (60 µL, 0.65 mmol), and the appropriate lipase were added to a 2 mL ampoule. The mixture ws stirred on a termo-shaker at 37 °C and 200 rpm, during the time period determined to each lipase. Assays using CAL-B, AK “Amano” 20 or PS “Amano” SD lasted 24 hours, while with AY “Amano” 30, and PPL were performed in 24, 72, 144, and 216 hours. After the predetermined preiod, the samples were filtered to remove the lipase before GC analysis^48^. Enantiomeric excesses and enantiomeric ratio were determined by enantioselective GC, as described above. Enantiomeric ratio was calculed based on the following equation:

$${\rm{E}}=\{\mathrm{ln}\,[e{e}_{{\rm{p}}}{\rm{x}}(1-e{e}_{{\rm{s}}})/(e{e}_{{\rm{p}}}+e{e}_{{\rm{s}}}]/\mathrm{ln}[e{e}_{{\rm{p}}}{\rm{x}}(1+e{e}_{{\rm{s}}})/(e{e}_{{\rm{p}}}+e{e}_{{\rm{s}}}]\},$$

where: *ee*_p_ = enantiomeric excess of the product, and *ee*_s_ = enantiomeric excess of the remaining alcohol^49^.

### Kinetic enzymatic resolution of cis-3 employing AK “AMANO” 20 – preparative scale

To a 125 mL Erlenmeyer, containing THF (32 mL), vinyl acetate (1920 µL, 20.8 mmol), and the lipase AK “AMANO” 20 (1.280 g), was added the alcohol *cis*-**3** (1.000 g, 6.4 mmol). The mixture was stirred on a rotatory shaker (37 °C, 150 rpm) during 24 hours. After this period, the lipase was filtered, and the solvent evaporated. The products were purified by flash chromatography (hexane:ethyl acetate - 2:1), yielding 1*R*,2*R*,6*S*-**3** (459 mg, 2.94 mmol, 46% yield) and 1*S*,2*S*,6*R*-**6** (562 mg, 2.84 mmol, 44.5% yield). 1*R*,2*R*,6*S*-**3** -[α]_D_ = +35.0 (c = 0.86, CHCl_3_, 24 °C) - *ee* = 98.8%, and 1*S*,2*S*,6*R*-6 - [α]_D_ = − 58.9 (c = 0.86, CHCl_3_, 24 °C) - *ee* = 75.7%^48^.

### Kinetic enzymatic resolution of cis-3 employing PS “AMANO” SD – preparative scale

To a 125 mL Erlenmeyer, containing TBME (32 mL), vinyl acetate (1920 µL, 20.8 mmol), and the lipase PS “Amano” SD (1.280 g), was added the alcohol *cis*-**3** (1.000 g, 6.4 mmol). The mixture was stirred on a rotatory shaker (37 °C, 150 rpm) during 24 hours. After this period, the lípase was filtered, and the solvent evaporated. The products were purified by flash chromatography (hexane:ethyl acetate - 2:1), yielding 1*R*,2*R*,6*S*-**3** (464 mg, 2.98 mmol, 46.6% yield) and 1*S*,2*S*,6*R*-**6** (566 mg, 2.86 mmol, 44.8% yield). 1*R*,2*R*,6*S*-**3** -[α]_D_ = +20.54 (c = 0.86, CHCl_3_, 24 °C) - *ee* = 64.2%, and 1*S*,2*S*,6*R*-**6** -[α]_D_ = − 70.23 (c = 0.86, CHCl_3_, 24 °C) - *ee* = 97.6%.

### (1*R*,2*R*,6*S*)-4,4,6-Trimethyl-7-oxabicyclo[4.1.0]heptan-2-yl acetate ((1*R*,2*R*,6*S*)-6)

To a magnetic stirred round bottom flask, containing pyridine (60 µL), (1*R*,2*R*,6*S*)-**3** (20 mg, 0.128 mmol), and dichloromethane (2 mL), was added Ac_2_O (60 µL, 0.65 mmol). The resulting mixture was stirred duritng a period of 12 hours at room temperature. The mixture was diluted with CH_2_Cl_2_ (5 mL) and the organic phase washed with saturated solution of NaHCO_3_, HCl 10%, and brine. The organic phase was dried with Na_2_SO_4_ and concentrated by vacuum. The crude product was purified by flash chromatography (hexane/ethyl acetate: 4/1), yielding (1*R*,2*R*,6*S*)-**6** (22 mg, 0.110 mmol, 85% yield). 1*R*,2*R*,6*S*-**6** -[α]_D_ = +74.9 (c = 0.86, CHCl_3_, 24 °C) - *ee* = 98.5%. ^*1*^*H NMR* (*200* *MHz*, *CDCl*_3_) *δ*: 0.87 (m, 3 H), 0.97–1.70 (m, 7 H), 2.03–2.07 (m, 3 H), 2.19 (s, 3 H), 3.09–3.16 (m, 1 H), 3.97–3.26 (m, 1 H). ^*13*^*C NMR* (*50* *MHz*, *CDCl*_3_) *δ*: 22.0, 24.5, 26.4, 31.0, 31.1, 35.6, 42.2, 58.9, 59.8, 69.9, 166.4. *MS* (*EI*, 70 *eV*)*: m/z* (*%*)*:* 156 (1), 140 (19), 138 (4), 123 (13), 109 (5), 107 (13), 100 (6), 99 (14), 98 (100), 97 (7), 95 (23), 93 (6), 91 (8), 85 (15), 83 (15), 82 (5), 81 (13), 79 (11), 77 (5), 71 (7), 69 (11), 67 (11), 57 (7), 55 (11), 53 (5), 43 (82), 41 (17).

### (1*S*,2*S*,6*R*)-4,4,6-Trimethyl-7-oxa-bicyclo[4.1.0]heptan-2-ol ((1*S*,2*S*,6*R*)-3)

A solution containing (1*S*,2*S*,6*R*)-**6** (200 mg, 1.01 mmol) in methanol (10 mL) was cooled to 5 °C and treated with NaOH (48 mg in 5 mL of methanol), maintaining the solution temperature under 15 °C during adding. The resulting mixture was stirred during 3 hours at 5 °C and during 12 additional hours at 25 °C. After this period, HCl (10 mL, 5% in water) was added and the reaction stirred at 25 °C during 10 minutes. Methanol was evaporated under reduced pressure and the aqueous solution extracted with dichloromethane (3 × 10 mL). The combined organic phase was washed with brine and dried with Na_2_SO_4_. The solvent was removed by vacuum and the crude product purified by flash chromatography (hexane/ethyl acetate: 2/1), yielding (1*S*,2*S*,6*R*)-**3** (145 mg, 0.93 mmol, 92% yield). (1*S*,2*S*,6*R*)-**3** −[α]_D_ = −34.5 (c = 0.86, CHCl_3_, 24 °C) - *ee* = 97.5%.

### 2-Hydroxy-3,5,5-trimethylcyclohex-2-enone (4)

A mixture containing epoxyisophorone (**1**) (0.5 g, 3.25 mmol), sulfuric acid (0.33 mL), acetic acid (0.66 mL), and sílica gel (4.00 g) in THF (25 mL) was prepared on a round bottom flask. The solvent of the resulting mixture was removed on a rotavapor at 60 °C. After complete removal of the solvent, the reaction was maintained on the rotavapor under the same temperature for additional 5 minutes. The resulting solid was then washed with dichloromethane, and the organic phase washed with aqueous Na_2_CO_3_ and brine and dried with sodium sulfate. The crude product was purified by flash chromatography (hexane/ethyl acetate: 2/1), yielding **4** (300 mg, 1.25 mmol, in 60% yield)^50^. ^*1*^*H NMR* (*200* *MHz*, *CDCl*_3_) *δ*: 1.07 (s, 6H), 1.89 (s, 3H), 2.26 (s, 2H), 2.36 (s, 2H), 6.05 (s, 1H). ^*13*^*C NMR* (*50* *MHz*, *CDCl*_3_) *δ*: 17.1, 28.3, 33.4, 44.6, 49.3, 127.8, 143.0, 194.0. *MS* (*EI*, *70* *eV*): *m/z* (*%*): 154 (M^+^, 70), 139 (29), 126 (10), 125 (13), 121 (9), 112 (23), 111 (25), 108 (6), 99 (5), 98 (46), 97 (8), 95 (4), 94 (5), 93 (10), 91 (5), 85 (8), 83 (25), 79 (4), 77 (6), 71 (10), 70 (100), 69 (19), 67 (7), 57 (17), 56 (5), 55 (38), 53 (7), 43 (25), 42 (9), 41 (28). *IR* (*ZnSe*, *ν*_*max*_): 1128, 1188, 1345, 1402, 1443, 1648, 1677, 2871, 2894, 2962, 3419 cm^−1^.

### (1*S*,6*S*)-4,4,6-Trimethyl-7-oxabicyclo[4.1.0]heptan-2-one ((1*S*,6*S*)-1)

A solution of (1*R*,2*R*,6*S*)-**3** (0.64 mmol) in dichloromethane (5 mL), was added to a suspension of PCC (1.56 mmol, 0.33 g), sodium acetate (0.32 mmol, 26 mg), and Celite® (347 mg) in dichloromethane (2 mL). The mixture was stirred at room temperature during 3 hours, followed by the addition of dry diethyl ether (10 mL). The resulting dark mixture was filtered trough a 1:1 mxture of Celite® and sílica flash which was washed with diethyl ether (2 × 5 mL). The crude product was concentrated by vacuum and purified by flash chromatography (hexane – ethyl acetate – 8:2), yielding (1*S*,6*S*)-**1** (0.52 mmol, 81 mg, *ee* = 87.5%, 82% yield)^51^.

### (1*R*,6*R*)-4,4,6-Trimethyl-7-oxabicyclo[4.1.0]heptan-2-one ((1*R*,6*R*)-1)

(1*R*,6*R*)-**1** was prepared following the same procedure described for (1*S*,6*S*)-**1**, starting with (1*S*,2*S*,6*R*)-**3** (100 mg, 0.64 mmol). (1*R*,6*R*)-**1** was obtained in 84% yield (0.54 mmol, 83 mg, *ee* = 89.3%).

### Field experiments

Field experiments where preformed in Mojú-PA, Brazil (1°55′S 48°45′W). Two sugarcane pieces (~10 cm) were placed inside of 2 L PET bottles. Pitfall^®^ traps were then placed on the top of the bottles. Ziploc® sachets (3 × 5 cm, 0.04 mm thickness, polyethylene) containing the different treatments were placed at the top of the traps. Eight different treatments were planned, with five repetitions each: **A**- *cis*-**3** (100 mg) + sugarcane, **B**- *trans*-**3** (100 mg) + sugarcane, **C**- Sugarcane (control), **D**- *cis*-**3** (100 mg) + *trans*-**3** (100 mg) + sugarcane, **E**- (1*R*,2*R*,6 *S*)-**3** (50 mg) + sugarcane, **F**- *cis*-**3** (100 mg) + **1** (10 mg) + **2** (10 mg) + **4** (10 mg) + sugarcane, **G**- 1 (200 mg) + sugarcane, **H**- 2 (200 mg) + sugarcane. Traps were randomly placed at the top of the coconut trees, with at least approximately 30 meters between then. Captured insects were counted every two days, during a period of six days.

## Supplementary information

Supporting Information

## References

[1] Goldstein AH, Galbally IE (2007). Known and unexplored organic constituents in the earth’s atmosphere. Environ. Sci. Technol..

[2] Payne, T. L. In *Pheromones* (ed M. Birch) 35–61 (American Elsevier 1974).

[3] Cardé RT (2014). Defining attraction and aggregation pheromones: teleological versus functional perspectives. J. Chem. Ecol..

[4] Landolt PJ (1997). Sex attractant and aggregation pheromones of male phytophagous Insects. Am. Entomol..

[5] Watanabe K, Shimizu N (2017). Identification of a sex pheromone of the chrysanthemum lace bug *Corythucha marmorata* (Hemiptera: Tingidae). Sci. Rep..

[6] Wen P (2017). The sex pheromone of a globally invasive honey bee predator, the Asian eusocial hornet, *Vespa velutina*. Sci. Rep..

[7] Xu T (2017). Identification of a male-produced sex-aggregation pheromone for a highly invasive cerambycid beetle, *Aromia bungii*. Sci. Rep..

[8] Yang S (2017). Male-produced aggregation pheromone of coffee bean weevil. Araecerus fasciculatus. J. Chem. Ecol..

[9] Ambrogi BG, Vidal DM, Zarbin PHG, Rosado-Neto GH (2009). Feromônios de agragação em Curculionidae (Insecta: Coleoptera) e sua implicação taxonômica. Quím. Nova.

[10] Ambrogi B, Zarbin P (2008). Aggregation pheromone in *Sternechus subsignatus* (Coleoptera: Curculionidae): olfactory behaviour and temporal pattern of emission. J. Appl. Entomol..

[11] Zarbin PH, Moreira MA, Haftmann J, Francke W, Oliveira AR (2007). Male-specific volatiles released by the brazilian papaya weevil, *Pseudopiazurus obesus*: partial identification and evidence of an aggregation pheromone. J. Braz. Chem. Soc..

[12] Oehlschlager AC (1992). Chirality and field activity of rhynchophorol, the aggregation pheromone of the American palm weevil. Naturwissenschaften.

[13] Vidal DM (2017). Male-specific volatiles released by *Homalinotus validus* (Coleoptera: Curculionidae) include (1*R*,2*S*)-grandisyl acetate, a new natural product. Tetrahedron Lett..

[14] Tumlinson J (1969). Sex pheromones produced by male boll weevil: isolation, identification, and synthesis. Science.

[15] Ramirez-Lucas P, Malosse C, Ducrot P-H, Lettere M, Zagatti P (1996). Chemical identification, electrophysiological and behavioral activities of the pheromone of *Metamasius hemipterus* (Coleoptera: Curculionidae). Bioorg. Med. Chem..

[16] Dhaliwal G, Vikas J, Dhawan A (2010). Insect pest problems and crop losses: changing trends. Indian J. Ecol..

[17] Vaurie P (1973). The weevil genera *Homalinotus* and *Ozopherus* of the Neotropical Cholinae (Coleoptera, Curculeonidae). B. Am. Mus. Nat. Hist..

[18] Ribeiro RC (2012). Damage by *Homalinotus depressus* in commercial coconut palm crops in the Amazonian region of Brazil. Phytoparasitica.

[19] Drmić Z (2017). Area‐wide mass trapping by pheromone‐based attractants for the control of sugar beet weevil (*Bothynoderes punctiventris* Germar, Coleoptera: Curculionidae). Pest Manag. Sci..

[20] El-Shafie H, Faleiro J, Al-Abbad A, Stoltman L, Mafra-Neto A (2011). Bait-free attract and kill technology (Hook™ RPW) to suppress red palm weevil, *Rhynchophorus ferrugineus* (Coleoptera: Curculionidae) in date palm. Fla. Entomol..

[21] Welter S (2005). Pheromone mating disruption offers selective management options for key pests. Calif. Agr..

[22] Vandendool H, Kratz PD (1963). A generalization of retention index system including linear temperature programmed gas-liquid partition chromatography. J. Chromatogr. A.

[23] Dostert K-H (2016). Adsorption of isophorone and trimethyl-cyclohexanone on Pd(111): A combination of infrared reflection absorption spectroscopy and density functional theory studies. Surf. Sci..

[24] Tellez MR, Schrader KK, Kobaisy M (2001). Volatile components of the cyanobacterium *Oscillatoria perornata* (Skuja). J. Agr. Food Chem..

[25] Eisner T, Hendry L, Peakall D, Meinwald J (1971). 2,5-Dichlorophenol (from ingested herbicide?) in defensive secretion of grasshopper. Science.

[26] Cloonan K, Bedoukian RH, Leal W (2013). Quasi-double-blind screening of semiochemicals for reducing navel orangeworm oviposition on almonds. PLOS One.

[27] Henbest, H. B., Meakins, G. D., Nicholls, B. & Taylor, K. J. Detection of the epoxide group by infrared spectroscopy. *J*. *Chem*. *Soc*., 1459–1462 (1957).

[28] Rissafi B (2001). Epoxyisophorone ring-opening: an efficient route for the introduction of functional groups at position 2 of isophorone. Tetrahedron.

[29] Marques FD, McElfresh JS, Millar JG (2000). Kovats retention indexes of monounsaturated C-12, C-14, and C-16 alcohols, acetates and aldehydes commonly found in lepidopteran pheromone blends. J. Braz. Chem. Soc..

[30] Cho BT, Kang SK, Kim MS, Ryu SR, An DK (2006). Solvent-free reduction of aldehydes and ketones using solid acid-activated sodium borohydride. Tetrahedron.

[31] Lattanzi, A., Iannece, P., Vicinanza, A. & Scettri, A. Renewable camphor-derived hydroperoxide: synthesis and use in the asymmetric epoxidation of allylic alcohols. *Chem*. *Commun*. 1440–1441 (2003). 12841281

[32] Mori K (2007). Significance of chirality in pheromone science. Bioorg. Med. Chem..

[33] Narasimhan S, Balakumar R (1998). Synthetic applications of zinc borohydride. Aldrichim. Acta.

[34] Payne JB (1973). Monoperphthalic acid. Organic Syntheses.

[35] Ye DY, Fringuelli F, Piermatti O, Pizzo F (1997). Highly diastereoselective epoxidation of cycloalkenols with monoperoxyphthalic acid in water. J. Org. Chem..

[36] Li, J. J. *Name Reactions: A Collection of detailed reaction mecanisms*. 3rd edn, (Springer 2006).

[37] Cooper MA, Ward AD (2004). Formation of dihydroxyselenides from allylic alcohols and their conversion to β-hydroxy epoxides via substitution of a phenylselenonyl group. Tetrahedron.

[38] Magnusson G, Thoren S (1973). New route to cyclopentene-1-carboxaldehydes by rearrangement of 2,3-epoxycyclohexanols. J. Org. Chem..

[39] D’Auria, M., Mauriello, G. & Rana, G. L. Volatile organic compounds from saffron. *Flavour Frag*. *J*. **19** (2004). 10.1093/chromsci/42.6.299 15296529

[40] Bianchi F, Mangia A, Mattarozzi M, Musci M (2011). Characterization of the volatile profile of thistle honey using headspace solid-phase microextraction and gas chromatography-mass spectrometry. Food Chem..

[41] Kus PM, Jerkovic I, Tuberoso CIG, Sarolic M (2013). The volatile profiles of a rare apple (*Malus domestica* Borkh.) honey: shikimic acid-pathway derivatives, terpenes, and others. Chem. Biodivers..

[42] Jerkovic I, Tuberoso CIG, Marijanovic Z, Kranjac M, Malenica-Staver M (2015). Antioxidant capacity and chemical profiles of *Satureja montana* L. honey: hotrienol and syringyl derivatives as biomarkers. Chem. Biodivers..

[43] Zarbin PHG, Ferreira JTB, Leal WS (1999). Metodologias gerais empregadas no isolamento e identificação estrutural de feromônios de insetos. Química Nova.

[44] Zarbin PHG, Lorini LM, Ambrogi BG, Vidal DM, Lima ER (2007). Sex pheromone of *Lonomia obliqua*: daily rhythm of production, identification, and synthesis. J. Chem. Ecol..

[45] Parra-Pedrazzoli AL (2006). Towards the identification and synthesis of the sex pheromone of the citrus leafminer, *Phyllocnistis citrella* stainton (Lepidoptera: Gracillariidae). Neotrop. Entomol..

[46] Armarego, W. L. F. & Chai, C. L. L. *Purification of laboratory chemicals*. 6th edn (Elsevier 2009).

[47] Nakata T, Tanaka T, Oishi T (1981). Highly stereoselective synthesis of erythro-α, β-epoxy alcohols by reduction of α, β-epoxy ketones with zinc borohydride. Tetrahedron Lett..

[48] Vidal DM, Fonseca MG, Zarbin PHG (2010). Enantioselective synthesis and absolute configuration of the sex pheromone of *Hedypathes betulinus* (Coleoptera: Cerambycidae). Tetrahedron Lett..

[49] Chen CS, Sih CJ (1989). General aspects and optimization of enantioselective biocatalysis in organic solvents: The use of lipases [new synthetic methods (76)]. Angew. Chem. Int. Ed..

[50] Zhu, R. *et al*. Practical preparation of diosphenols by ring opening of α,β-epoxyketones catalyzed by silica gel supported acids. *Synlett*, 2267–2271 (2007).

[51] Carruthers, W. & Coldham, I. *Modern methods of organic synthesis*. 4th edn, (Cambridge 2004).

