# Supplementary material for: Isophorone derivatives as a new structural motif of aggregation pheromones in Curculionidae
Source: Sci Rep. 2019 Jan 28;9:776. doi: 10.1038/s41598-018-37156-8 (PMC6349938; doi:10.1038/s41598-018-37156-8)

Supplementary information for:

Isophorone Derivatives as a New Structural Motif of Aggregation Pheromones in Curculionidae

Diogo Montes Vidal,[a,b] Marcos Antonio Barbosa Moreira,[a,c] Miryan Denise Araujo Coracini,[a,d] and Paulo Henrique Gorgatti Zarbin[*,a]

[a] Departamento de Química - Laboratório de Semioquímicos, Universidade Federal do Paraná, Curitiba – PR – Brazil, Francisco H. dos Santos, 100 - 81531-980.
e-mail: pzarbin@ufpr.br.

[b] Departamento de Química, Universidade Federal de Minas Gerais, Belo Horizonte - MG – Brazil.

[c] Embrapa Tabuleiros Costeiros, Aracaju - SE – Brazil.

[d] Centro de Ciências Biológicas e da Saúde, Universidade Estadual do Oeste do Paraná, Cascavel – PR – Brazil.

Content

1- Relative configuration of natural homalinol (3) 2

2- Absolute configuration of natural epoxyisophorone (1) 3

3- Enzymatic kinetic resolution of *cis*-3 4

4- Y-tube olfactometer bioassays employing synthetic homalinol (3) 5

5- Field experiments 6

6 SPECTRA 7

1- Relative configuration of natural homalinol (3)

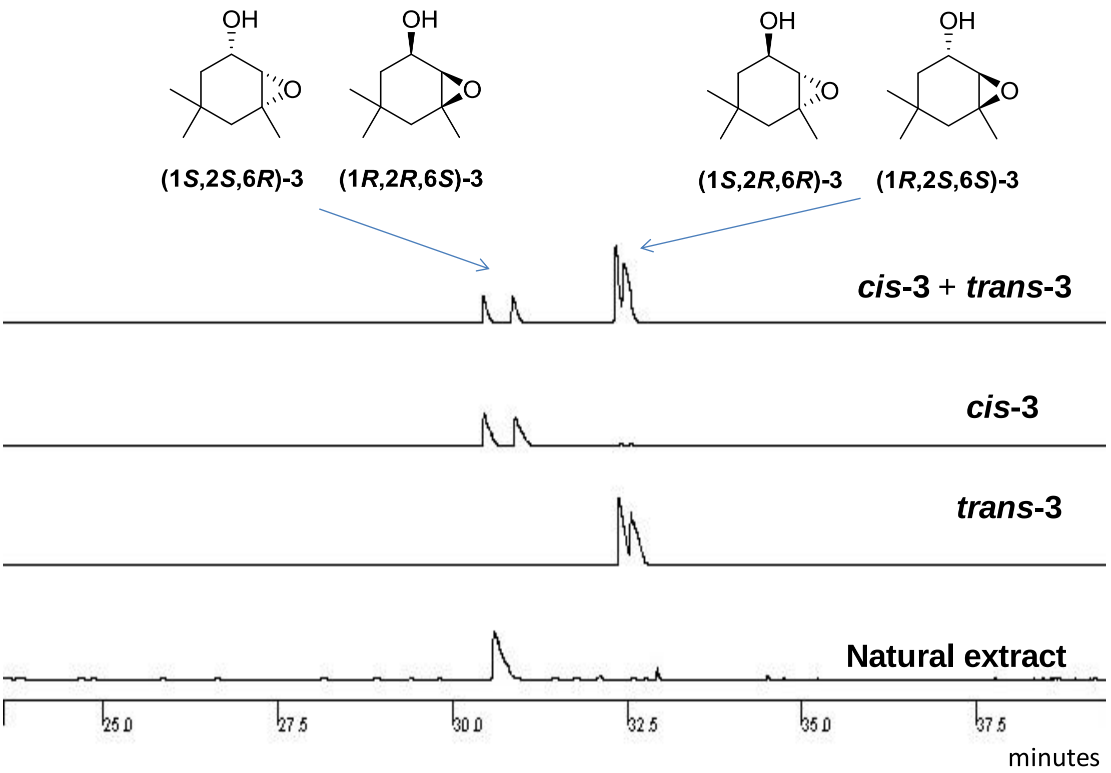

Figure S1. Determination of relative configuration of the major *H. depressus* pheromone component by retention time comparison of mixtures of stereoisomers of homalinol (**3**) and the natural extract (GC column: β-DEX 325®, Supelco).

2- **Absolute configuration of natural epoxyisophorone (1)**

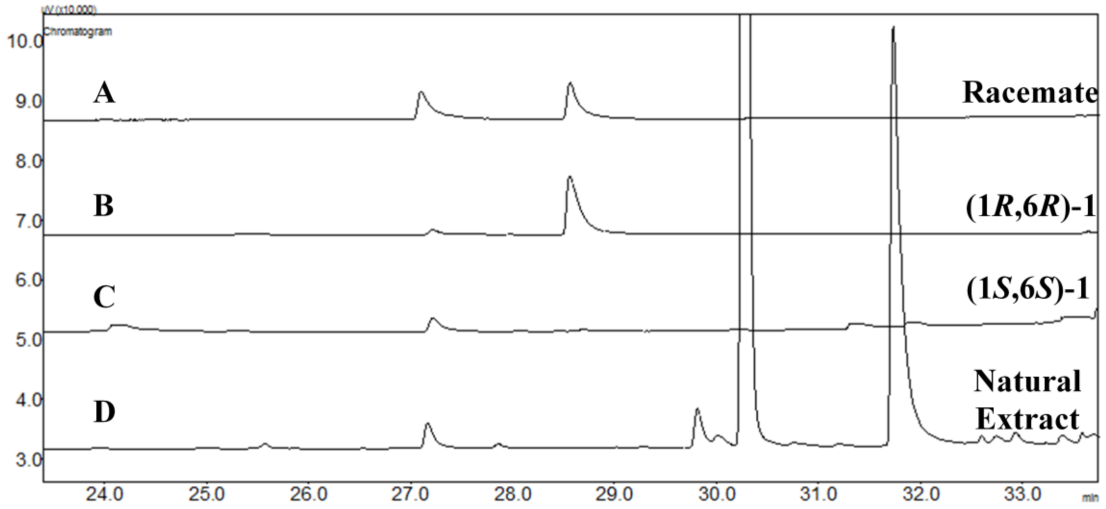

Figure S2. Determination of absolute configuration of natural epoxyisophorone (**1**) by retention time comparison of the racemate, (1*S*,6*S*)-**1**, (1*R*,6*R*)-**1**, and the natural extract (GC column: β-DEX 325®, Supelco).

3- Enzymatic kinetic resolution of *cis*-3

Table S1. Kinetic resolution of *cis*-3, using PS "Amano" SD or AK "Amano" 20, and different organic solvents and reaction times.

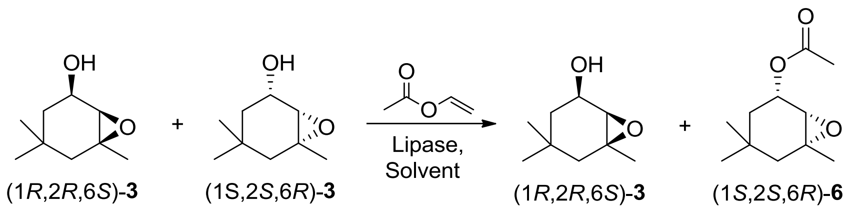

Lipase	Solvent	Time (h)	ee_s[a] (%)	ee_p[a] (%)	c[b] (%)	E[c]
**PS "Amano" SD**	**Hexane**	**24**	24,6	98,8	19,9	>200
	**THF**	**24**	51,9	95,2	35,3	68
	**TBME**	**24**	65,4	98,6	39,9	>200
	**Vinyl acetate**	**24**	12,5	94,0	11,7	37
**AK "Amano" 20**	**Hexane**	**24**	44,0	95,7	31,5	71
	**THF**	**24**	99,9	80,0	55,5	65
	**TBME**	**24**	99,9	66,8	59,9	36
	**Vinyl acetate**	**24**	99,9	82,5	54,8	82

Conditions: 37 °C, 200 rpm, *cis*-**3** (30 µL, 0.17 mmol), organic solvent (1 mL), vinyl acetate (60 µL, 0.65 mmol), lipase (40 mg);

[a] *ee* = enantiomeric excess – calculated by comparison of the GC peak areas of the respective enantiomers: ee_x=100x(A_M-A_m)/(A_M+A_m), where A_M= peak area of major enantiomer and A_m= peak area of minor enantiomer.

[b] c (conversion) = [ee_s/(ee_s+ee_p)]x100

[c] E= {ln[ee_px(1-ee_s)/(ee_p+ee_s]/ln[ee_px(1+ee_s)/(ee_p+ee_s]}

4- Y-tube olfactometer bioassays employing synthetic homalinol (3)

Table S2. Number of males and females of *Homalinotus depressus* attracted do different odor sources on Y-tube olfactometer.

.

Assay	Sex	Odor	N total[1]	N response[2]	Control[3]	Treatment[4]
**A**	Females	*cis*-**3** x hexane	39	30	9 (30 %) b[5]	21 (70 %) a
**B**	Females	*trans*-**3** x hexane	39	30	16 (53 %) a	14 (47 %) a
**C**	Females	*cis* + *trans*-**3** x hexane	36	30	14 (47 %) a	16 (53 %) a
**D**	Males	*cis*-**3** x hexane	37	30	7 (23 %) b	23 (77 %) a
**E**	Males	*trans*-**3** x hexane	38	30	8 (27 %) b	22 (73 %) a
**F**	Males	*cis* + *trans*-**3** x hexane	31	30	10 (33 %) b	20 (67 %) a

[1] Total number of insects used on the bioassay. [2] Number of insects that responded to the bioassay. [3] Hexane was used as control. [4] Solutions of the interest compounds in hexane. [5] Values on the same line, followed by the same letter do not differ statistically (Qui-square, p<0,05).

5- Field experiments

Table S3: Treatments employed on field bioassays.

Treatment	Lure
**A**	***cis*-3** (100 mg) **+** sugarcane
**B**	***trans*-3** (100 mg) **+** sugarcane
**C**	**Control**[1] + sugarcane
**D**	***cis*-3** (100 mg) + ***trans*-3** (100 mg) + sugarcane
**E**	***R*,*R*,*S*-3** (50 mg) + sugarcane
**F**	***cis*-3** (100 mg)+ **1** (10mg)+ **2** (10mg)+ **4** (10 mg)
**G**	**1** (200 mg)
**H**	***2*** (200 mg)

[1] Treatment indicated as control used just the sachet as lure, without pheromonal components.

6 SPECTRA

6.1 ^{1}H SPECTRUM FOR COMPOUND 1

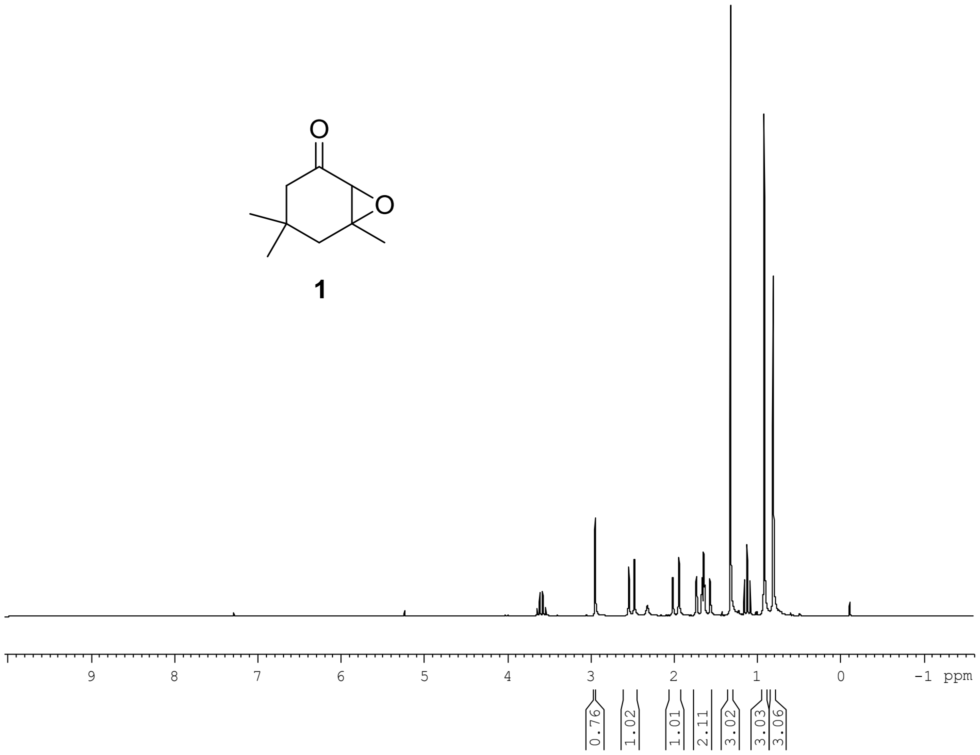

6.2 ^{13}C AND DEPT 135 NMR SPECTRA FOR COMPOUND 1

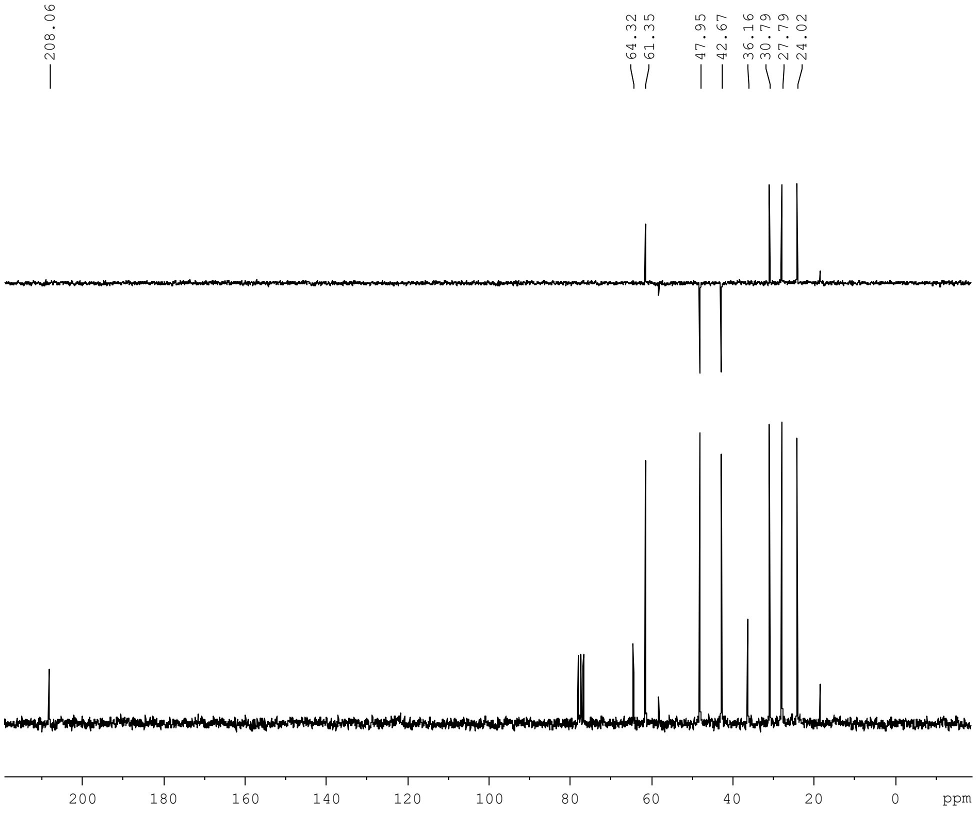

6.3 ^{1}H SPECTRUM FOR COMPOUND *trans*-3

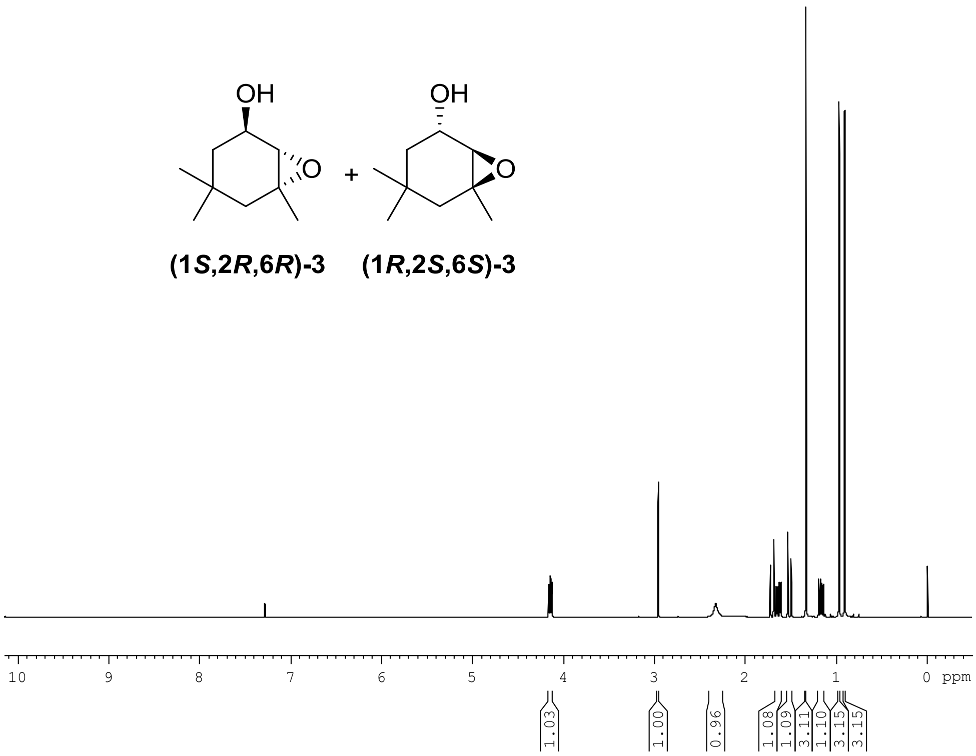

6.4 ^{13}C AND DEPT 135 NMR SPECTRA FOR COMPOUND *trans*-3

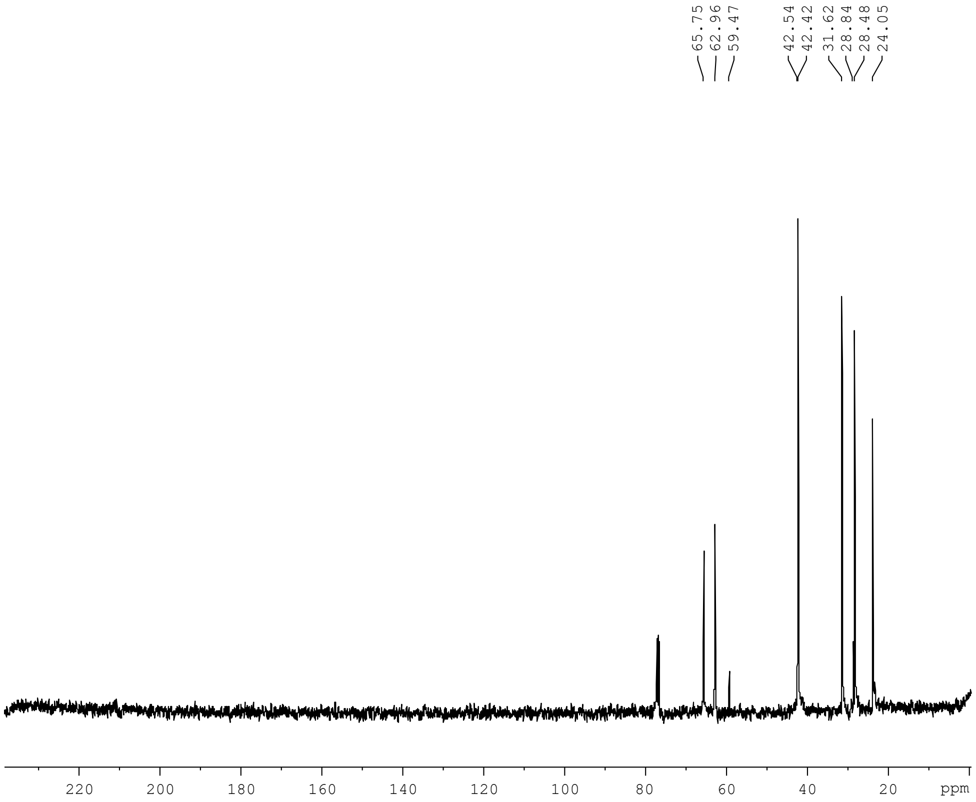

6.5 ^{1}H SPECTRUM FOR COMPOUND *cis*-3

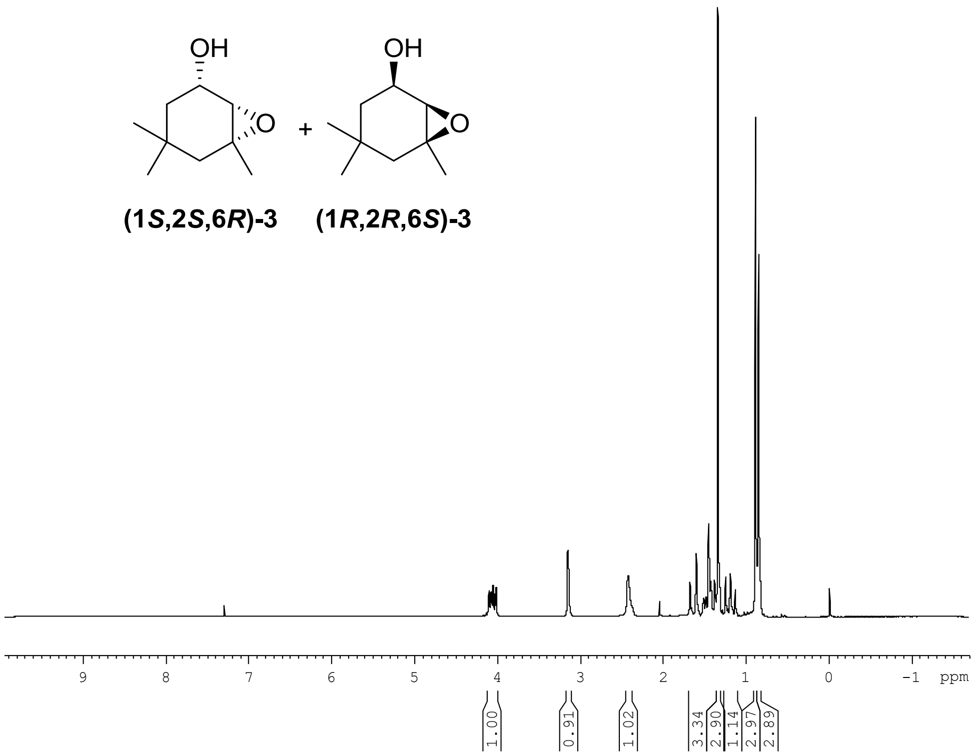

6.6 ^{13}C AND DEPT 135 NMR SPECTRA FOR COMPOUND *cis*-3

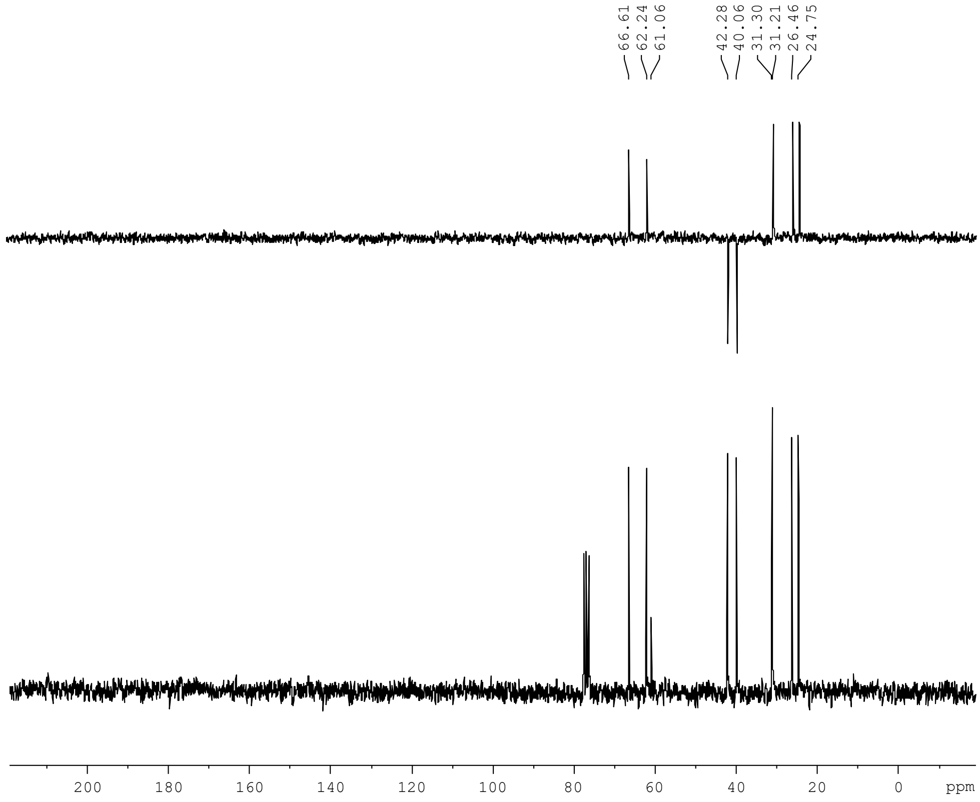

6.7 ^{1}H SPECTRUM FOR COMPOUND 4

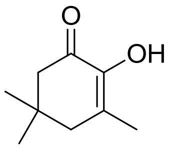

4

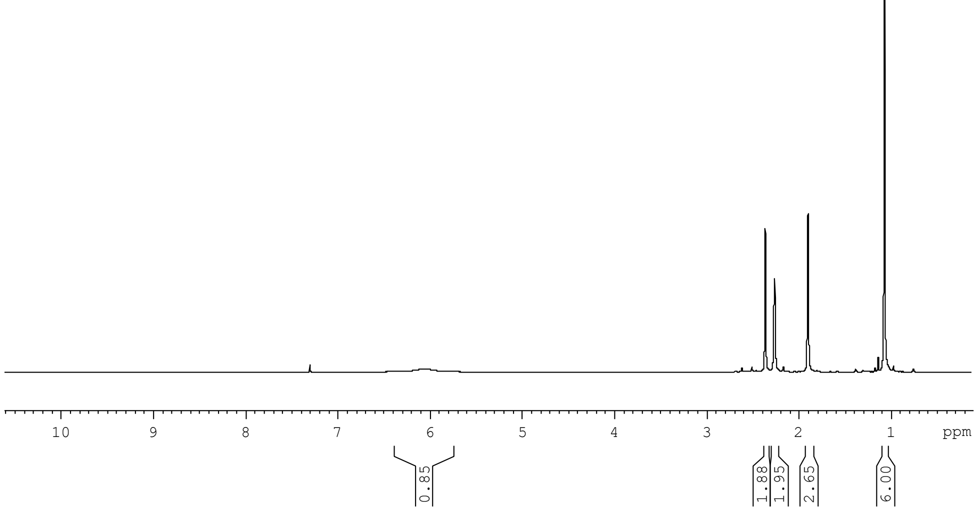

6.8 ^{13}C AND DEPT 135 NMR SPECTRA FOR COMPOUND 4

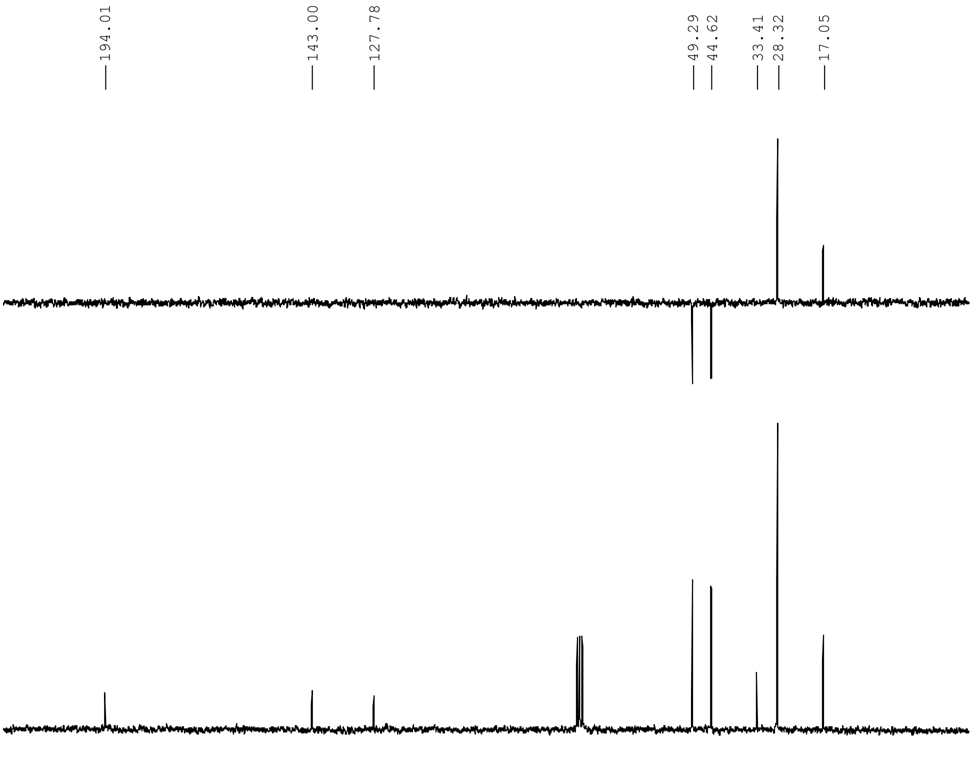

6.9 ^{1}H SPECTRUM FOR COMPOUND 5

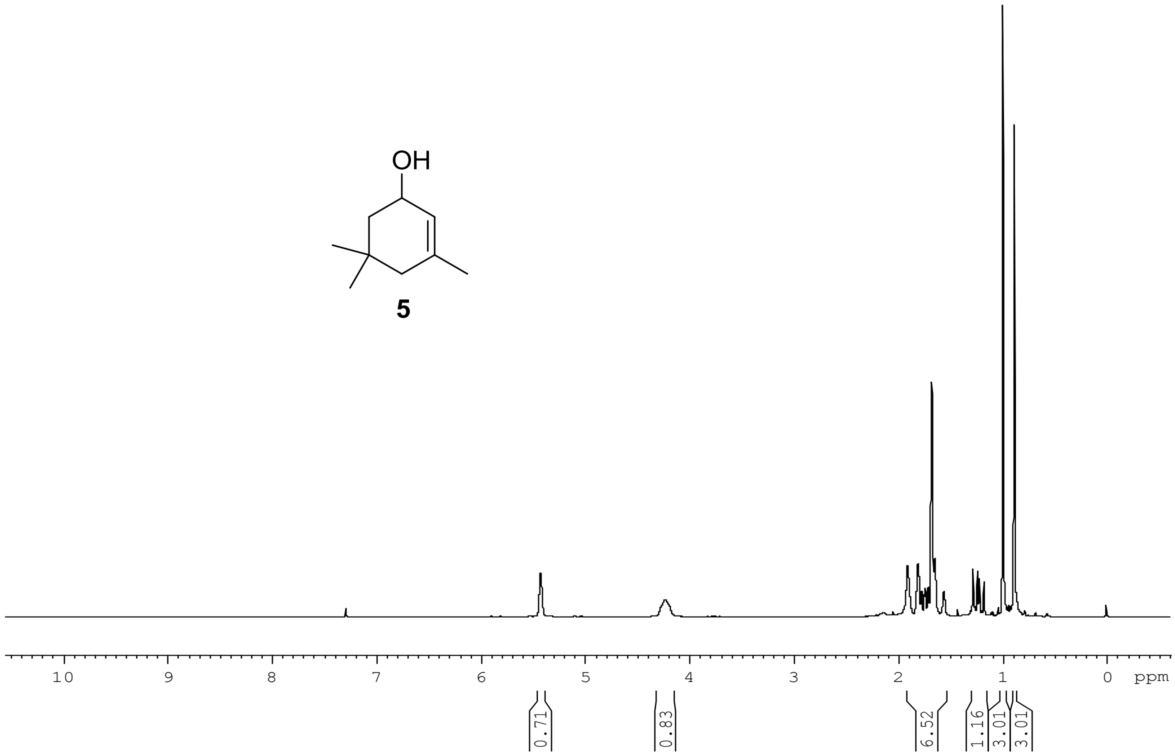

6.10 ^{13}C AND DEPT 135 NMR SPECTRA FOR COMPOUND 5

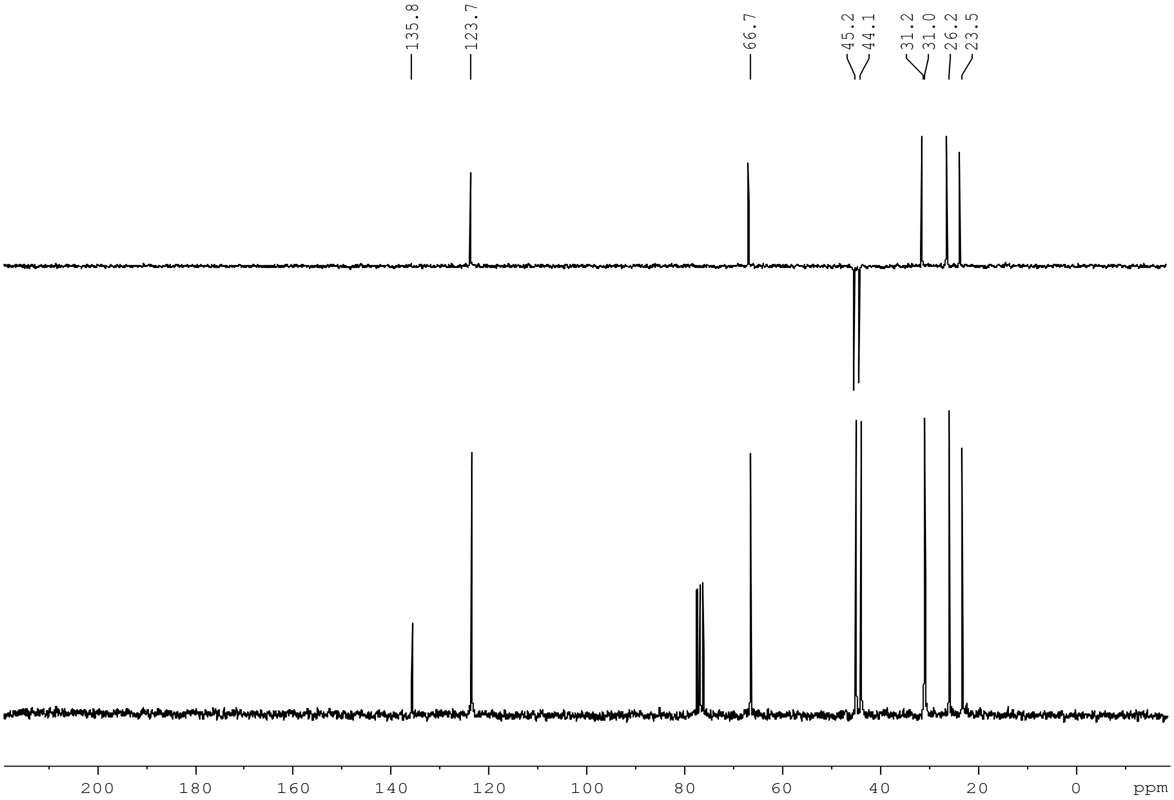

6.11 MASS SPECTRUM FOR COMPOUND 5

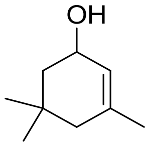

5

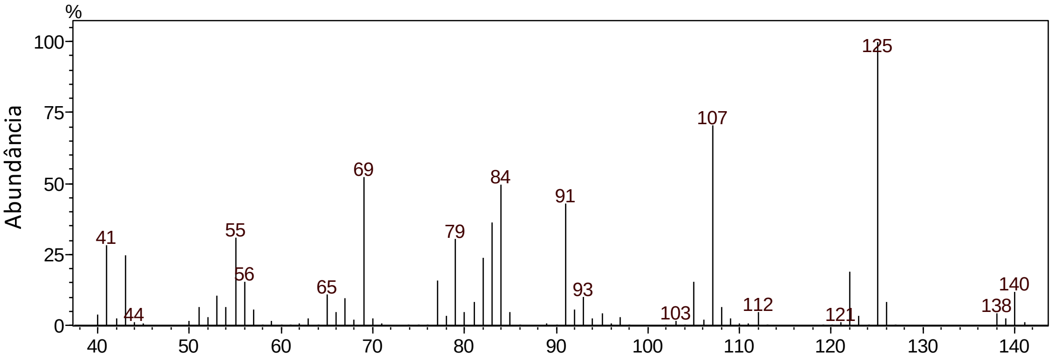

6.12 MASS SPECTRUM FOR COMPOUND (1*R*,2*R*,6*S*)-6

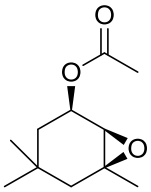

198,26g.mol^{-1}

(1*R*,2*R*,6*S*)-6

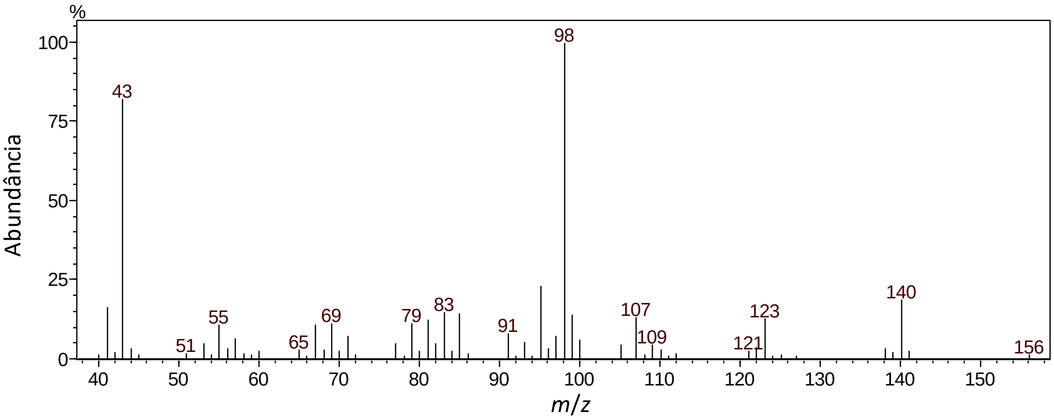

Supplement: Supplementary file 1 — Supporting Information [file 41598_2018_37156_MOESM1_ESM.pdf]
